# Immunotoxicity of Four Per- and Polyfluoroalkyl Substances Following 28-Day Oral Repeat Dosing in Rats Assessed by the Anti-Sheep Red Blood Cell IgM Response

**DOI:** 10.3390/toxics13060490

**Published:** 2025-06-10

**Authors:** Michael F. Hughes, Michael J. DeVito, Grace Patlewicz, Russell S. Thomas, Linda D. Adams, Jeffrey L. Ambroso, Xi Yang, Bindu G. Upadhyay, Stefanie C. M. Burleson, Elaina M. Kenyon

**Affiliations:** 1 Center for Computational Toxicology and Exposure, Office of Research and Development, U.S. Environmental Protection Agency, Durham, NC 27709, USA; devito.michael@epa.gov (M.J.D.); patlewicz.grace@epa.gov (G.P.); thomas.russell@epa.gov (R.S.T.); adams.lindad@epa.gov (L.D.A.); kenyon.elaina@epa.gov (E.M.K.); 2 RTI International, Durham, NC 27709, USA; jambroso@rti.org (J.L.A.); xyang@rti.org (X.Y.); bupadhyay@rti.org (B.G.U.); 3 Burleson Research Technologies, Morrisville, NC 27560, USA; sburleson@brt-lab.com

**Keywords:** perfluoroalkyl substance, polyfluoroalkyl substance, immunotoxicity, repeat dose, IgM, oral, T cell-dependent antibody response

## Abstract

Some PFASs are immunotoxic in rodent models and associated with diminished vaccine response in exposed humans. This study assessed the immunotoxicity of four PFASs via the T cell-dependent IgM antibody response (TDAR) to sheep red blood cells (SRBCs) in adult male rats following 28-day oral repeat dosing. The PFASs included 1H,1H,9H-perfluorononyl acrylate (PFNAC), 1H,1H,2H,2H-perfluorohexyl iodide (PFHI), 2-chlorotetrafluoropropionic acid (CTFPA), and 3,3,4,4,5,5,5-heptafluoropentan-2-one (MHFPK), administered in corn oil. The positive control was cyclophosphamide (CPS). Rats were dosed with vehicle or PFAS from Days 0 to 27. On Day 22, an immunogenic dose of SRBCs was administered intravenously. Positive control animals were administered CPS by intraperitoneal injection from Days 22–27. On Day 28, the animals were euthanized; blood, thymus, and spleen samples were collected and weighed. Serum IgM was quantified by enzyme-linked immunosorbent assay. Body weights were unaffected in PFAS-treated rats, except for 3 and 10 mg/kg/day PFNAC-treated rats on Days 24, 27, and 28. Relative spleen and thymus weights and serum IgM levels were not affected by the PFASs at the doses tested, whereas CPS-treated animals had significant decreases in these parameters. The rat TDAR, as assessed by the anti-SRBC IgM response, was not affected by these four PFAS test agents following a 28-day oral exposure.

## 1. Introduction

The manufacture of per- and polyfluoroalkyl substances (PFASs), their use in industrial and commercial products, and their disposal practices have led to their contamination of the environment and detection in human tissues [1,2]. Many PFASs are persistent in the environment and biological systems because of their physicochemical properties. The C-F bond, which is the hallmark of the PFAS structure, is highly resistant to abiotic and biotic degradation. The resultant outcome has been the detection of PFASs in a variety of environmental and biological matrices, such as water, freshwater fish, and human serum. In tap water collected in Southeast Los Angeles, California, between 2020 and 2021, the levels of perfluorooctanoic acid (PFOA), perfluorooctane sulfonic acid (PFOS), and perfluorohexane sulfonic acid (PFHxS) among samples with detectable concentrations ranged from 6.8 to 13.6, 9.4 to 17.8, and 5.0 to 5.2 ng/L, respectively [3]. In tissues of smallmouth bass from the mid-Atlantic area in the United States collected during 2014–2019, the levels of PFOA, PFOS, and PFHxS were below detection–4.5, 5.4–6000, and below detection–4.5 ng/g, respectively [4]. The Centers for Disease Control (CDC) reported geometric mean levels of PFOA, PFOS, and PFHxS at 1.42, 4.25, and 1.08 µg/L in human serum of the United States population collected between 2017 and 2018 [5].

There is a concern that exposure to PFASs may result in hepatotoxicity, immunotoxicity, cancer, and other chronic diseases [2], which may be due in part to the biological persistence of these contaminants in humans. Olsen et al. [6] reported that in retired fluorochemical production workers, the serum elimination half-lives for PFOA, PFOS, and PFHxS were 3.8, 5.4, and 8.5 years, respectively. Regarding the immunotoxicity of PFASs, the National Toxicology Program (NTP) conducted a systematic review of the immunotoxicity of PFOA and PFOS [7]. They concluded that there was a high level of evidence (and confidence) in the animal data and a moderate level of evidence (and confidence) in the human data for immunosuppression of the antibody response following exposure to PFOA and PFOS. An example of immunosuppression in animals is the significant reduction in sheep red blood cell (SRBC)-specific serum IgM antibody levels and relative weights of the spleen and thymus in mice exposed to PFOA (0–30 mg/kg/day) for 10 or 15 days [8]. In humans, a two-fold increase in serum concentration of PFOA and PFOS in a pediatric population was associated with a decreased antibody concentration to the tetanus and diphtheria vaccines [9]. However, whether PFASs are generally immunotoxic is still under debate. There are animal studies showing no or limited immunotoxic effects of PFASs. Pierpoint et al. [10] administered PFOS (potassium salt) to mice by oral gavage for 28 days at environmentally relevant doses (0.15–50 µg/kg). No effects were observed on B cells, T cells, granulocytes from several organs, serum antibodies, or several serum cytokines. Garvey et al. [11] used a weight-of-evidence approach to evaluate whether PFASs are immunotoxic. Their conclusions were that there is only moderate evidence, mostly from animal studies, supporting the hypothesis that PFOS and PFOA are immunotoxic. Antoniou et al. [12] also questioned the relevance of an immunotoxic effect of PFASs, particularly with the high doses required in animals to elicit an effect on the immune system.

**Figure 1:**
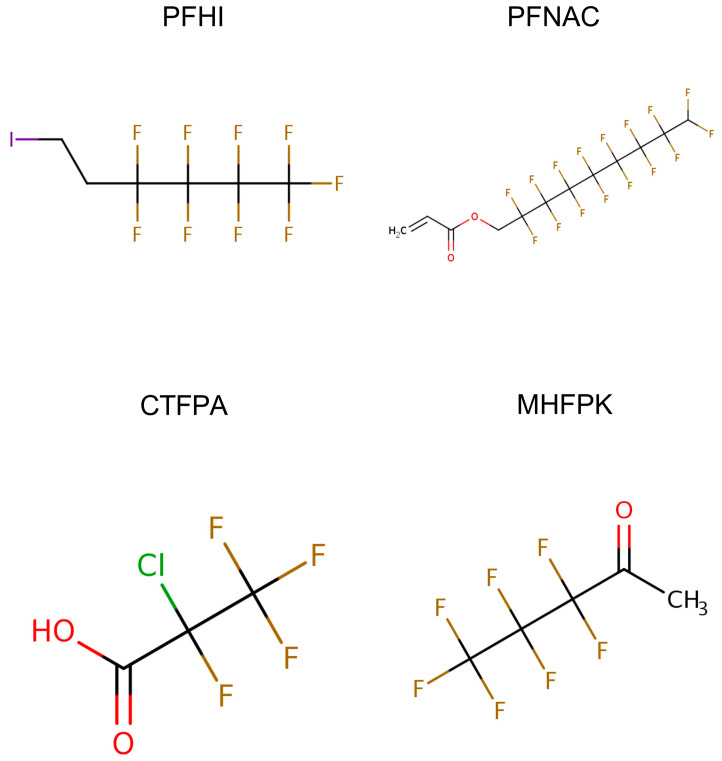
Structure of 1H,1H,9H-perfluoronyl acrylate (PFNAC), 1H,1H,2H,2H-perfluorohexyliodide (PFHI), 2-chlorotetrafluoropropionic acid (CTFPA), and 3,3,4,4,5,5,5-heptafluoropentan-2-one (MHFPK). The figures are from the US EPA CompTox Chemicals Dashboard (https://comptox.epa.gov/dashboard/, accessed on 22 May 2025).

The objective of this study was to evaluate the immunotoxicity via the T cell-dependent antibody response (TDAR) to SRBCs in male rats following 28-day oral repeat dosing to four selected data-poor PFASs, for which no repeat dose toxicity information is available. These PFASs were also selected to represent prioritized data-poor chemical categories of PFASs, as described in Patlewicz et al. [13]. The chemicals included 1H,1H,9H-perfluorononyl acrylate (PFNAC), 1H,1H,2H,2H-perfluorohexyl iodide (PFHI), 2-chlorotetrafluoropropionic acid (CTFPA), and 3,3,4,4,5,5,5-heptafluoropentan-2-one (MHFPK) (Figure 1, Table 1). These PFAS chemicals are not presently listed in the CDC’s Data Tables for Environmental Chemicals, so the extent of human exposure or their presence in the environment is not clear [5]. However, several national and international public health agencies have published these chemicals on lists of chemicals of concern, for example, the Contaminant Candidate List 5 (PFAS subset) of the US Environmental Protection Agency (US EPA), because of their presence in public water systems. PFHI is listed on the US EPA’s Toxic Substance Control Act’s active substance list and the Minnesota Department of Health Chemicals of High Concern and Priority Chemicals; PFNAC and MHFPK are on the European Inventory of Existing Commercial Chemicals, the PFASs: List from the Swedish Chemicals Agency (KEMI) Report, and the Organization of Economic and Cooperative Development’s New Comprehensive Global Database of Per- and Polyfluoroalkyl Substances (PFASs) list. These lists and their descriptions can be obtained from the US EPA Comptox Chemicals Dashboard (https://comptox.epa.gov/dashboard/, accessed on 22 May 2025). The TDAR assay is considered an apical guideline for immunotoxicity studies, as the effect of a chemical on several immune cell types, such as B cells and T-helper cells, can be detected. In addition, the response to SRBCs, which is a very sensitive endpoint, involves multiple components of the immune system [14,15]. As such, the study we conducted will provide information on immunosuppression for data-poor PFASs using standard animal studies for this purpose.

## 2. Materials and Methods

### 2.1. Chemicals

Four PFAS chemicals were assessed for immunotoxicity, including 1H,1H,9H-perfluorononyl acrylate (PFNAC), 1H,1H,2H,2H-perfluorohexyl iodide (PFHI), 2-chlorotetrafluoropropionic acid (CTFPA), and 3,3,4,4,5,5,5-heptafluoropentan-2-one (MHFPK) (Table 1, Figure 1). The chemicals were purchased from Synquest Laboratories, Inc. (Alachua, FL, USA). Their purity was ≥97%. Corn oil was purchased from Welch, Holme & Clark Company (Newark, NJ, USA). Details regarding the analysis of the four PFAS test agents and corn oil are described in Kenyon et al. [16]. The positive control cyclophosphamide (CPS) monohydrate was purchased from Sigma Aldrich (St. Louis, MO, USA). Sheep red blood cells (SRBCs) in Alsever’s solution were from Colorado Serum Company (Denver, CO, USA).

### 2.2. Animals

Male Sprague Dawley (SD^®^ IGS) rats were purchased from Charles River Laboratories (Raleigh, NC, USA). The rats were housed in a facility accredited by the Association for Assessment and Accreditation of Laboratory Animal Care. All procedures with animals were reviewed by the Institutional Animal Care and Use Committee (IACUC) of Burleson Research Technologies (Morrisville, NC, USA). The animals were received at 10 weeks for the SRBC concentration optimization study and 5–7 weeks of age for the 28-day oral repeat dosing immunotoxicity study. The animals were acclimated 7–10 days prior to use. The animals were housed in ventilated shoebox-style plastic cages with BioFresh (Lab Supply, Durham, NC, USA) bedding, with 3 animals housed in each cage. Nylon bones and rat retreats were provided as enrichment. The animals were provided Purina 5002 Rodent Chow (LabDiet, Richmond, IN, USA) and tap water ad libitum. The housing rooms were maintained on 12 h light/dark cycles, temperatures of 22 ± 3 °C, and a relative humidity range of 30–70%. The animals were randomized into study groups with the criterion that all study animals were within 20% of the mean body weight at study commencement.

### 2.3. Sheep Red Blood Cell Optimization Study

This phase of this study determined the optimal SRBC dose and blood collection time point for the 28-day oral repeat dosing immunotoxicity study. The animals (N = 9/SRBC dose) were monitored once daily over the course of this study for clinical appearance, including body condition, coat appearance, posture, and lethargy. Body weights were determined during randomization, within one day of dosing. At the commencement of this study, the weight variation of the rats used was within 20% of the mean weight of all the study animals.

SRBCs were suspended in sterile phosphate-buffered saline at three different concentrations (1 × 10^8^, 2 × 10^8^, and 4 × 10^8^ SRBC/mL) and 0.5 mL was administered intravenously via the tail vein on Day 0 to immunize animals (induction of a T cell-dependent antibody response). The SRBC doses and blood collection time points tested are listed in Table 2.

On Days 4 and 5, 0.2 mL of blood was collected via tail vein puncture into serum separator tubes. The blood was allowed to clot at room temperature for 30–60 min, and the tubes were centrifuged at 1300× *g* at room temperature for 10 min. Duplicate aliquots of serum (≥50 µL) were taken and stored at ≤ −70 °C until being analyzed for anti-SRBC IgM. On Day 6, all surviving animals were weighed and euthanized with CO_2_. A maximum amount of blood was collected via the inferior vena cava of each animal into a serum separator collection tube. The serum was collected and handled as previously described. The spleens were collected and weighed.

IgM in serum was quantified using an ELISA kit from Life Diagnostics (West Chester, PA, USA). Briefly, the diluted test samples and standards were added to microwells and incubated for 45 min. The wells were washed, and horse radish peroxidase-conjugated anti-rat IgM was added to the wells. The microplate was incubated at room temperature for 45 min, the wells were washed, and the substrate solution was added. Color development was ceased after 20 min by the addition of the stop solution. The optical density was determined spectrophotometrically at 450 nm. Samples and standards were run in duplicate.

### 2.4. Twenty-Eight-Day Oral Repeat Exposure to PFASs and Evaluation of the T Cell-Dependent Antibody Response

**Figure 2:**
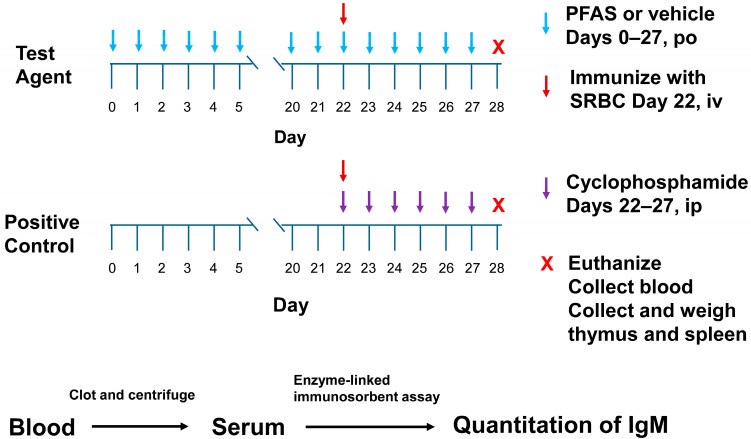
Experimental workflow of the 28-day oral repeat dose immunotoxicity assay.

This study followed the methods outlined in the US EPA Health Effects Test Guideline OPPTS 870.7800 Immunotoxicity [17]. The studies were performed in a block design, wherein each block used one test chemical along with positive and vehicle control groups. A workflow of the experimental assay is shown in Figure 2. Male rats (N = 12/dose group) were dosed daily with vehicle or a PFAS test chemical via oral gavage on Days 0–27 (28 consecutive days) (Table 1). (Note, in the 200 mg/kg/day PFHI dose group, N = 11. One rat in this group was mis-dosed over the course of 4 days. It was decided to remove this animal from this study.) The test guideline states that either sex may be used in this assay. It further states that if one sex is more sensitive or believed to be more sensitive, then that sex should be used. For PFASs, there is a general trend indicating that male rodents are more sensitive than female rodents to the toxic effects of these chemicals. Based on this trend, male rats were chosen as the test model. The administered doses were as follows: PFHI, 0, 12.5, 25, 50, 100, and 200 mg/kg/day; PFNAC, 0, 0.1, 0.3, 1, 3, and 10 mg/kg/day; CTFPA, 0, 1.9, 3.8, 7.5, 15, and 30 mg/kg/day; MHFPK, 0, 18.8, 37.5, 75, 150, and 300 mg/kg/day. The doses were based on previous 14-day oral repeat dose range-finding studies [16]. Although there were no effects on survival or body weight of the male rats administered the 4 PFASs over the course of 14 days, dose-dependent increases in liver weight were observed for PFNAC, PFHI, and CTFPA. Individual dose volumes were calculated based on the weekly body weight by each animal. Test substance formulations were stirred for at least 30 min prior to dosing and continuously during dosing. The positive control group was treated once daily with 15 mg/kg cyclophosphamide monohydrate in saline via intraperitoneal injection on Days 22–27. SRBCs were suspended in sterile phosphate-buffered saline and administered intravenously via the tail vein on Day 22 in a volume of 0.5 mL at a concentration of 4 × 10^8^ SRBCs/mL (dose of 2 × 10^8^ SRBCs/rat). Test article and cyclophosphamide were administered after immunization with the SRBCs.

The animals were observed twice daily for clinical signs of toxicity. They were monitored for clinical appearance, including body condition, coat appearance, posture, and lethargy. Body weights were recorded twice weekly, including within one day of the commencement of this study and just prior to euthanasia. At the commencement of this study, the weight variation in the animals assigned was within 20–30% of the mean weight of all study animals.

Beginning on Day 0, the feed consumption was determined once each week by adding pre-weighed rat diet to each respective food container. If a supplemental diet was required during a week, the mass of the supplemented diet was added to the weekly totals for those cages. The feed remaining in each container at the end of the monitoring period was weighed. Weekly mean feed consumption for each rat (obtained by dividing feed consumption for each respective cage by the number of rats in that cage; 3 animals/cage) was calculated and reported.

On Day 28, all surviving animals were weighed and euthanized by CO_2_ asphyxiation. Blood was collected by cardiac puncture or from the inferior vena cava into serum separator collection tubes. The blood was processed and stored as previously described. The spleen and thymus from each animal were collected and weighed.

Serum samples were analyzed for anti-SRBC IgM using a commercial ELISA kit, as previously described.

### 2.5. Data Analysis

The data are presented as mean ± standard deviation (SD). The data were analyzed by Analysis of Variance testing, and two general linear models were fit using SAS (v. 9.2 or later, Cary, NC, USA). Statistically significant levels were set at *p* < 0.05. A vehicle versus positive control (cyclophosphamide) model and a vehicle versus test substance model were tested. The parameters tested were feed consumption, weekly and terminal body weights, organ weights (absolute and relative), and anti-SRBC IgM. A Dunnett’s test was performed on the least square means to test for treatment differences from the control. This method allowed for multiple testing while maintaining an experiment-wide alpha error of 0.05.

## 3. Results

### 3.1. SRBC Optimization

Clinical observations of the rats in the SRBC optimization study showed normal responses to the treatment, except for hair loss in a few animals. All rats, other than two, gained weight over the course of the experiment. One rat lost 3 g and a second lost 6 g over the course of this study. On Day 6, the body weight of the 0.5 × 10^8^ SRBC/rat group was significantly greater (*p* < 0.05) than the 1 × 10^8^ SRBC/rat group. Spleen weights were not affected by the dose of SRBCs (Supplemental Information Tables S1 and S2, respectively). The serum IgM levels were significantly greater on Days 5 (*p* < 0.05) and 6 (*p* < 0.01) than the levels on Day 4. Overall, the highest levels of serum IgM were approximately 50,000 units/mL in the 2 × 10^8^ SRBC/rat group on Day 6 (Table S3). A dose of 2 × 10^8^ SRBC/rat was chosen as the dose for immunization in the PFAS study.

### 3.2. Animal Observations of PFAS-Treated Rats

Animal observations were generally normal throughout this study, apart from some hair loss and/or minor scratches documented for a few study animals. One animal in the 25 mg/kg/day PFHI group had necrosis on the tip of the tail due to an injury of unknown origin on Days 17–28. Overall, these observations were considered unrelated to treatment or study activities.

### 3.3. Body Weight of PFAS-Treated Rats

**Figure 3:**
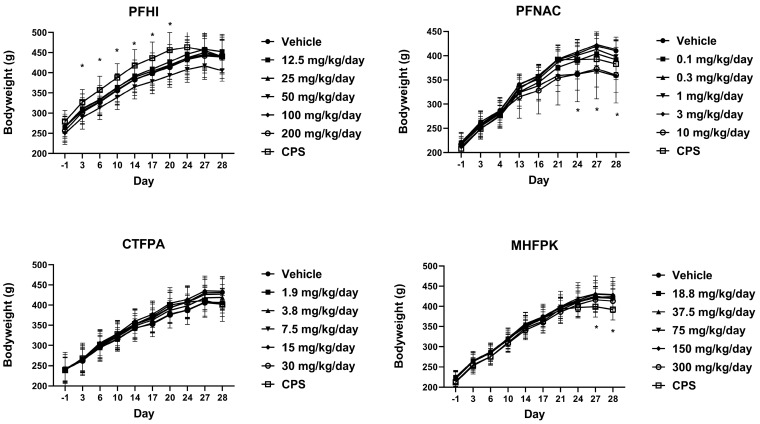
Mean group body weights of rats dosed by oral gavage daily from Day 0 to 27 with PFHI, PFNAC, CTFPA, or MHFPK. The vehicle control was corn oil. The positive control rats were administered CPS in sterile saline by intraperitoneal injection on Days 22–27. The rats were administered the antigen SRBC iv on Day 22. The rats were euthanized on Day 28. The data represent mean ± SD, N = 12/dose group, except for the 200 mg/kg/day PFHI dose group, N = 11. * Significantly different from the vehicle control, *p* < 0.05.

Mean group body weights (Figure 3) were generally similar between the PFAS-treated and vehicle control groups. There were some reductions in body weight at 50 mg/kg/day PFHI on Days 17–28, but these were ≤ 8% compared to the control and not significant. Statistically significant reductions were observed at 3 mg/kg/day PFNAC on Days 24, 27, and 28. The body weights at 10 mg/kg/day PFNAC on these days were similarly reduced but were not significant compared to the control (<13% for both doses). CPS-positive control body weights generally plateaued following Day 24, unlike the trending increases observed for the vehicle control. In the CPS-treated rats in the PFHI group, the body weights were significantly elevated from Day 3 to Day 20. However, this is not a treatment-related effect because these animals did not receive CPS until Day 22. There were no differences in body weight from the control in this group following the administration of CPS. Significant reductions in the body weight of the CPS-treated animals of the MHFPK study were observed on Days 27 and 28.

### 3.4. Feed Consumption of PFAS-Treated Rats

**Figure 4:**
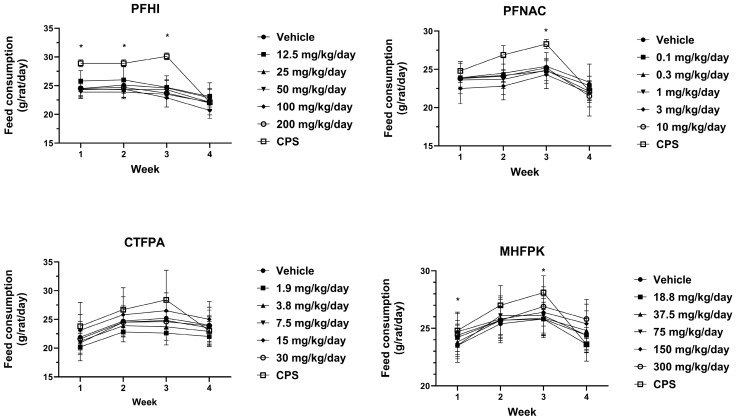
Mean feed consumption of rats dosed by oral gavage daily from Day 0 to 27 with PFHI, PFNAC, CTFPA, or MHFPK. The vehicle control was corn oil. The positive control rats were administered CPS in sterile saline by intraperitoneal injection on Days 22–28. The rats were administered the antigen SRBC iv on Day 22. The rats were euthanized on Day 28. The data represent mean ± SD, N = 12/dose group, except for the 200 mg/kg/day PFHI dose group, N = 11. * Significantly different from vehicle control, *p* < 0.05.

Mean feed consumption values (Figure 4) were generally consistent between the PFAS-treated and vehicle control groups. For PFNAC, the lowest mean feed consumption was observed at 0.1 mg/kg/day during the last week of this study. Other values were consistent between the PFNAC dose groups. In all studies, the CPS-positive control group mean feed consumption increased relative to the vehicle control through Week 3. Note that the rats had not been treated with CPS up to this time. The week 4 CPS mean feed consumption values obtained following the initiation of dosing were decreased relative to the previous Week 3 values but were consistent with the Week 4 vehicle feed consumption values.

### 3.5. Spleen Weights of PFAS-Treated Rats

**Figure 5:**
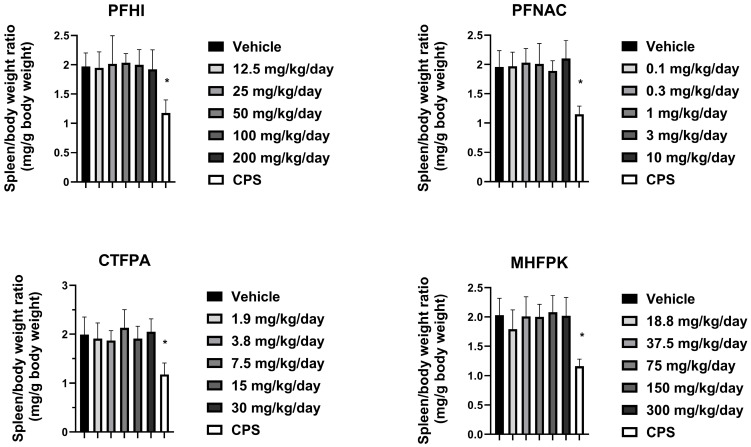
Spleen/body weight ratio of rats dosed by oral gavage daily from Day 0 to 27 with PFHI, PFNAC, CTFPA, or MHFPK. The vehicle control was corn oil. The positive control rats were administered CPS in sterile saline by intraperitoneal injection on Days 22–28. The rats were administered the antigen SRBC iv on Day 22. The rats were euthanized on Day 28. The data represent mean ± SD, N = 12/dose group, except for the 200 mg/kg/day PFHI dose group, N = 11. * Significantly different from the vehicle control, *p* < 0.05.

The effect of the PFAS compounds on the spleen was assessed by determining spleen/body weight ratios (i.e., relative weight) (Figure 5). The spleen/body weight ratios of the PFAS-treated animals were generally consistent with their respective vehicle control group. There was a minor decrease in the 3 mg/kg/day PFNAC-treated group, but this is not considered test substance-related because of the small decrease and lack of a dose response. The CPS-positive control spleen/body weight ratios significantly decreased compared to the vehicle control in all test groups, as expected. The absolute spleen weights are shown in Figure S1. No significant effects on the spleen weight from the PFAS treatment were observed.

### 3.6. Thymus Weights of PFAS-Treated Rats

**Figure 6:**
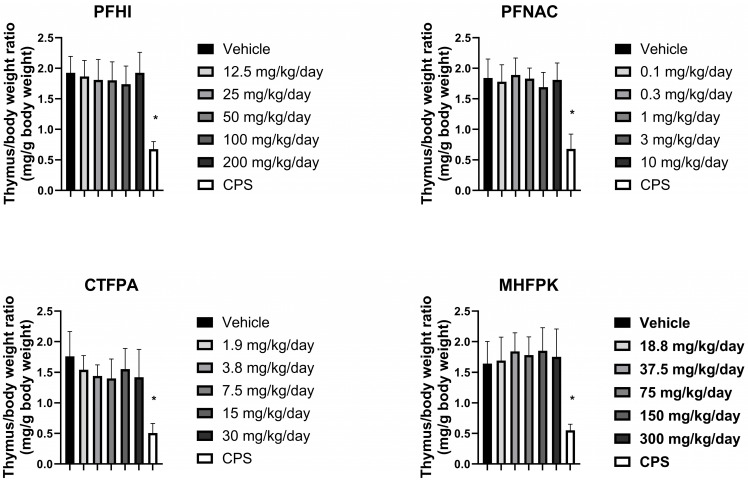
Thymus/body weight ratio of rats dosed by oral gavage daily from Day 0 to 27 with PFHI, PFNAC, CTFPA, or MHFPK. The vehicle control was corn oil. The positive control rats were administered CPS in sterile saline by intraperitoneal injection on Days 22–28. The rats were administered the antigen SRBC iv on Day 22. The rats were euthanized on Day 28. The data represent mean ± SD, N = 12/dose group, except for the 200 mg/kg/day PFHI dose group, N = 11. * Significantly different from the vehicle control, *p* < 0.05.

The effect of the PFAS compounds on the thymus was assessed by determining thymus/body weight ratios (i.e., relative weight) (Figure 6). The ratios of the PFAS-treated animals were consistent with their respective vehicle control group. A minor reduction was observed at 50 mg/kg/day PFHI and 3 mg/kg/day PFNAC. These decreases were not considered test-substance-related based on the minor level of decrease observed and lack of a dose response. As expected, the thymus/body weight ratio of the cyclophosphamide-positive-control-treated rats was significantly decreased compared to the vehicle control in all the groups. The absolute weights of the thymus are shown in Figure S2. A significant decrease in thymus weight was observed for PFNAC at 3 mg/kg/day and CTFPA at 3.8 and 7.5 mg/kg/day (Supplemental Figure S2). An approximate 20% decrease compared to the vehicle control was observed at these doses. Significant effects on the absolute thymus weights were not observed at higher doses for these two PFAS compounds or for PFHI and MHFPK.

### 3.7. Anti-SRBC IgM of PFAS-Treated Rats

**Figure 7:**
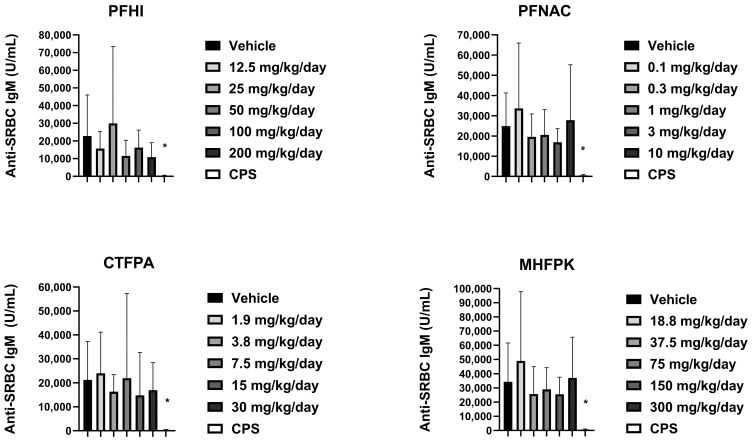
Serum anti-SRBC IgM response of rats dosed by oral gavage daily from Day 0 to 27 with PFHI, PFNAC, MHFPK, or CTFPA. The vehicle control was corn oil. The positive control rats were administered CPS in sterile saline by intraperitoneal injection on Days 22–28. The rats were administered the antigen SRBC iv on Day 22. The rats were euthanized on Day 28. The data represent mean ± SD, N = 12/dose group, except for the 200 mg/kg/day PFHI dose group, N = 11. * Significantly different from vehicle control, *p* < 0.05.

The mean anti-SRBC IgM values were generally consistent between the vehicle control and PFAS-treated rats (Figure 7). A slight reduction was observed at 50 and 100 mg/kg/day PFHI. There was a minor decrease at 3 mg/kg/day PFNAC. These minor decreases were not considered test-substance-related findings given the variability observed and lack of a dose response. A significant reduction in anti-SRBC IgM relative to the vehicle control was observed in the CPS-positive control animals in the four study groups. Based on the results of this study, the no-observed adverse effect level (NOAEL) for immunotoxicity in male rats is the highest doses tested, i.e., 10 mg/kg/day for PFNAC, 200 mg/kg/day for PFHI, 30 mg/kg/day for CTFPA, and 300 mg/kg/day for MHFPK.

## 4. Discussion

PFASs are environmental contaminants, and human exposure to these highly fluorinated and persistent substances occurs daily. Some PFASs have immunosuppressive properties in rodents and potentially in humans. We conducted an immunotoxicology guideline study in rats, which tested a 28-day oral repeat exposure to four selected PFASs, namely, PFNAC, PFHI, CTFPA, and MHFPK. Several apical endpoints were measured. The main endpoint evaluated, i.e., serum levels of IgM formed in response to the antigen SRBCs, was not affected following exposure to these four PFASs at the doses administered. The relative weights of the spleen and thymus, and the absolute weight of the spleen, were also not affected by this exposure. The absolute weight of the thymus was affected at mid-dose by CTFPA and PFNAC but was not dose-responsive, suggesting the decrease was not chemically related. A positive immunosuppressive control, i.e., CPS, was included in this study. The animals responded to CPS as expected, with significantly reduced spleen and thymus weights and serum levels of IgM, indicating the test system was reacting properly.

The NTP published two 28-day oral toxicity studies in rats with seven PFASs, consisting of four carboxylates and three sulfonates [18,19]. These were not immunotoxicity studies but general toxicology studies. They reported decreases in body weight with 6 of the compounds and decreases in spleen and thymus weights for some of the chemicals. Decreased spleen weight was observed for the carboxylates PFOA, perfluorononanoic acid (PFNA), and perfluorodecanoic acid (PFDA). Perfluorohexanoic acid (PFHxA) had no effect on spleen weight. For the sulfonates, PFOS decreased spleen weight, but perfluorobutane sulfonic acid (PFBS) and perfluorohexane sulfonate potassium salt (PFHxSK) had no effect. Decreased thymus weight was observed for all the carboxylates (PFHxA, PFNA, PFOA, PFDA) and two of the sulfonates, i.e., PFBS and PFOS. PFHxSK had no effect on thymus weight. The decreases in body weight may have had a role in the observed spleen and thymus weights. PFHxSK was the only PFAS in the NTP reports that did not affect body weight or the weights of the thymus and spleen. In our study, body weights were slightly but significantly affected by PFNAC, but the effect was not dose-dependent. PFHI, MHFPK, and CTFPA had no significant effect on body weight. The spleen and thymus relative weights were not affected by PFAS treatment. Only the absolute weight of the thymus was affected by CTFPA and PFNAC, but the effect was not dose-dependent. The NTP studies and the present study suggest that a change in organ weight is related to a change in body weight following chemical exposure. However, the change in organ weight may be related to the extent of body weight change, for example, greater than 20%. Dose and length of exposure may also have a role in this effect.

Although the immunosuppressive response to PFASs in rodents, particularly PFOS and PFOA in mice, is well established [8,20,21], the lack of an immunotoxic response under our study conditions indicates that not all PFASs elicit this type of effect. The lack of an immunosuppressive effect of the chemicals in our study may be related to test species, sex, administered dose, chemical disposition, protein binding, or other factors.

There are other studies that show a minimal immunosuppressive effect, or a lack thereof, of PFASs in rodents. Loveless et al. [22] orally dosed male CD rats and male CD-1 mice with PFOA (ammonium salt; 0–30 mg/kg/day) for 29 days. They reported no immune-related changes in treated rats, including serum IgM levels in response to SRBCs. In contrast, significant immune-related changes were observed in mice, but only at doses that produced significant systemic toxicity. In Loveless et al. [22], liver/body weight ratios increased about 2-fold in treated rats (at 10, 30, and 300 mg/kg/day), but in treated mice at similar doses, this same ratio was elevated about 4-fold. Pierpoint et al. [10] administered PFOA (potassium salt) to female and male C57BL/6 mice by oral gavage for 28 days at environmentally relevant doses (0–50 µg/kg/day). No effects were observed on B cells, T cells, granulocytes isolated from several organs, or serum antibodies and cytokines examined. Qazi et al. [23] exposed male B6C3F1 mice to PFOS (ammonium salt) in the diet for 28 days. The intake was approximately 250 µg PFOS/kg/day. Decreased body weight gain and increased liver weight of the treated mice were reported. However, no effects on several immunological factors were reported, including cellular compositions of the thymus and spleen and serum levels of IgM and IgG towards SRBCs. In male and female Sprague Dawley rats exposed by Lefebvre et al. [24] to PFOS (potassium salt, 2–100 mg/kg) in their diet for 28 days, significant body weight reductions were observed in both sexes at 50 and 100 mg/kg PFOS. A significant increase in spleen weight relative to body weight was observed in female rats at 100 mg/kg PFOS. However, there were no histological changes in the spleen of these rats. Females at 2 mg/kg and males at 20 mg/kg PFOS had significant increases in liver/body weight ratios. In males, there was a significant increase in serum total IgG, IgG2a, and IgG2c with increased dose. In contrast, IgG1 levels in male rats decreased significantly at 2 and 20 mg/kg PFOS, while serum total IgM levels were not affected. In female rats, total serum IgM and IgG2 levels were significantly increased at 100 mg/kg PFOS. Frawley et al. [25] observed limited immunotoxic effects of perfluoro-*n*-decanoic acid administered by the oral route to female Harlan Sprague Dawley rats (0–2 mg/kg/day, 28 days) and female B6C3F1/N mice (0–5 mg/kg/week, 4 weeks). Because of treatment-related toxicity, data from rats at 1 and 2 mg/kg/day were not included in the analysis. Our results with PFNAC, PFHI, CTFPA, and MHFPK show a lack of an immunotoxic effect of the four PFASs tested in rats.

The reason for the lack of an immunotoxic effect for the four tested PFASs is not known; however, there may be several potential explanations. Toxicokinetics involves the processes of absorption, distribution, metabolism, and excretion of a chemical and may have a role in the responses observed in our study. While there is limited disposition data for the individual PFASs tested in the present study, we recently conducted 90-day oral repeat dosing studies with these same chemicals and doses, which show effects on liver, kidney, and clinical chemistry, indicating that these PFASs are absorbed systemically and distributed to tissues [16]. Plasma from the male rats collected on Day 91 and analyzed for the parent showed 0.05 µg/mL for PFHI at 200 mg/kg/day and 84.4 µg/mL for CTFPA at 30 mg/kg/day. For PFNAC, the parent was not detected, but one large peak was detected, which may be a metabolite. Plasma levels for MHFPK were not determined because an analysis method could not be developed [16]. Thus, the lack of an immunotoxic effect in the present study seems unlikely to be due to limited absorption of the four PFASs tested, though the bioavailability of the compounds may be important. Pizzuro et al. [26] reviewed the available PFAS toxicokinetic data and reported that rats absorb PFOA and PFOS to an estimated percentage exceeding 90%. The tissue distribution of PFOA and PFASs in rats can be dose- and sex-dependent, but they are principally detected in the liver, kidney, and serum [26]. Metabolism of many PFASs, particularly substances such as PFOA and PFOS, is minimal because of the strength of the C-F bond [1]. Crizer et al. [27] screened 54 PFASs for substrate depletion using human hepatocytes. Substrate depletion was not detected for 35 of the PFASs tested. PFAS fluorotelomers and fluorosulfonamides were the substances that were more readily metabolized by the hepatocytes. Fluorotelomers are perfluoroalkyl substances, of which one or more carbons are bonded to hydrogen, not fluorine. PFAS fluorotelomers have been noted to be metabolized by rats [28]. PFNAC and PFHI are perfluoroalkyl substances and may be more likely to be metabolized relative to CTFPA and MHFPK. However, it is not known whether PFNAC or PFHI were metabolized to products that were not immunotoxic in this study. Excretion has an important role in the elimination of PFASs, as many of these compounds are not metabolized. The excretion of PFASs is dependent on the chemical, species, and sex [26]. The elimination half-lives in rats for several PFASs are measured in hours or days as opposed to days or years in humans. For example, the elimination half-life of PFOA in the female rat ranges from 1.9 to 4.6 hr, but in humans, it ranges from 2.3 to 8.5 years [26]. It is possible that the PFASs tested in our study were eliminated rapidly enough to prevent an immune response.

It is well documented that rodents exhibit immunosuppressive effects when exposed to PFASs [7,29]. However, many of these studies were conducted with mice and at relatively high doses of PFOA or PFOS [8,20,22]. A wide variety of endpoints following exposure to PFASs in rodents have been examined, such as the weight of spleen and thymus, splenic and thymic cellularity, and serum levels of histamine and tumor necrosis-α, but few studies have reported on what is considered the most sensitive endpoint: SRBC-specific IgM antibody response [30,31,32,33]. Antoniou et al. [12] commented on the species difference between mice and rats in the potency of PFASs, as well as the observation that the interaction of peroxisome proliferator-activated receptor-alpha with PFOS may have a role in the immunosuppressive effect in mice, as reported by Qazi et al. [34]. The species differences and other evidence have led several scientists to question whether PFASs are immunosuppressive in humans and to call for additional research [11,12].

## 5. Conclusions

The four PFASs tested in a 28-day repeat oral immunotoxicity study (PFNAC, PFHI, CTFPA, and MHFPK) were not immunosuppressive in male rats based on thymus and spleen weights and serum IgM levels in response to immunization with the antigen SRBCs. Although some PFASs are known immunotoxicants in rodents, particularly mice, the negative immunotoxicity results for these four PFASs add to the body of literature to help inform the chemical structural, toxicokinetic, and toxicodynamic determinants of immunotoxicity for this class of compounds.

## Figures and Tables

**Table 1:** PFASs tested in the 28-day oral repeat dose immunotoxicity study.

PFAS	CASRN ^a^	DTXSID ^b^	Purity	Dose (mg/kg/day)
1H,1H,9H-perfluorononyl acrylate (PFNAC)	4180-26-1	DTXSID00194615	97%	0, 0.1, 0.3, 1.0, 3.0, 10
1H,1H,2H,2H-perfluorohexyl iodide (PFHI)	2043-55-2	DTXSID1047578	99%	0, 12.5, 25, 50, 100, 200
2-chloro-2,3,3,3-tetrafluoropropionic acid (CTFPA)	6189-02-2	DTXSID30476698	99%	0, 1.9, 3.8, 7.5, 15, 30
3,3,4,4,5,5,5-heptafluoropentan-2-one (MHFPK)	355-17-9	DTXSID00188993	99%	0, 18.8, 37.5, 75, 150, 300

^a^ Chemical Abstract Services Registry Number; ^b^ Distributed Structure-Searchable Toxicity Substance Identifier.

**Table 2:** Optimization of doses of SRBCs administered to rats and time points of blood collection.

Group	Time Point ^1^	SRBC Concentration	SRBC Dose ^2^	Number of Male Rats
1	Day 4/5/6	1 × 10^8^ cells/ml	0.5 × 10^8^ cells/rat	9
2	Day 4/5/6	2 × 10^8^ cells/ml	1 × 10^8^ cells/rat	9
3	Day 4/5/6	4 × 10^8^ cells/ml	2 × 10^8^ cells/rat	9

^1^ Days 4 and 5 were in-life blood sample collections. Day 6 was a terminal collection. ^2^ SRBCs were administered on Day 0 to all animals in a volume of 0.5 mL via intravenous administration.

## Data Availability

The data are available at https://doi.org/10.23645/epacomptox.29067014.

## Supplementary Materials

The following supporting information can be downloaded at: https://www.mdpi.com/article/10.3390/toxics13060490/s1, Figure S1. Spleen weight (g) of rats dosed by oral gavage daily from Day 0 to 27 with PFHI, PFNAC, CTFPA, or MHFPK; Figure S2. Thymus weight (g) of rats dosed by oral gavage daily from Day 0 to 27 with PFHI, PFNAC, CTFPA, or MHFPK; Table S1. Male body weights pre- and post-administration of sheep red blood cells (SRBCs); Table S2. Male spleen weights from the sheep red blood cell (SRBC) optimization study; Table S3. IgM (Units/mL) in male rat serum following exposure to sheep red blood cells (SRBCs).

## Abbreviations

The following abbreviations are used in this manuscript:

SRBCs	sheep red blood cells
PFNAC	1H,1H,9H-perfluorononyl acrylate
PFHI	1H,1H,2H,2H-perfluorohexyl iodide
CTFPA	2-chloro-2,3,3,3-tetrafluoropropionic acid
MHFPK	3,3,4,4,5,5,5-heptafluoropentan-2-one
CPS	cyclophosphamide
CDC	Centers for Disease Control
PFAS	per- or polyfluoroalkyl substance
PFOA	perfluorooctanoic acid
PFOS	perfluorooctane sulfonic acid
PFHxS	perfluorohexane sulfonic acid
PFBS	perfluorobutane sulfonic acid
PFNA	perfluorononanoic acid
PFDA	perfluorodecanoic acid
PFHxA	perfluorohexanoic acid
PFHxSK	perfluorohexane sulfonate potassium salt
TDAR	T cell-dependent antibody response
NTP	National Toxicology Program
US EPA	United States Environmental Protection Agency
