# Supplementary material for: Immunotoxicity of Four Per- and Polyfluoroalkyl Substances Following 28-Day Oral Repeat Dosing in Rats Assessed by the Anti-Sheep Red Blood Cell IgM Response
Source: Toxics. 2025 Jun 10;13(6):490. doi: 10.3390/toxics13060490 (PMC12197378; doi:10.3390/toxics13060490)
Supplement: Supplementary file 1 [file toxics-13-00490-s001.zip › toxics-3611440-supplementary.pdf]

Supplemental Information

# Immunotoxicity of four per- and polyfluoroalkyl substances following 28-day oral repeat dosing in rats assessed by the anti-sheep red blood cell IgM response

Michael F. Hughes[1], Michael J. DeVito[1], Grace Patlewicz[1], Russell S. Thomas[1], Linda D. Adams[1], Jeffrey L. Ambroso[2], Xi Yang[2], Bindu G. Upadhyay[2], Stefanie C.M. Burleson[3], Elaina M. Kenyon[1]

[1]Center for Computational Toxicology and Exposure, Office of Research and Development, U.S. Environmental Protection Agency, Research Triangle Park, NC

[2]RTI International, Research Triangle Park, NC

[3]Burleson Research Technologies, Morrisville, NC

Table S1 Male body weights pre- and post-administration of sheep red blood cells (SRBC). Rats were administered SRBC on Day 0.

| Dose (SRBC/rat) | Day -9 | Day -1 | Day 6 |
|---|---|---|---|
| $0.5 \times 10^8$ | 273.9 ± 10.3[a] g | 354.3 ± 21.1 g | 376.8 ± 30.4[b] g |
| $1 \times 10^8$ | 256.9 ± 9.6 g | 328.1 ± 19.2 g | 344.1 ± 25.5 g |
| $2 \times 10^8$ | 268.3 ± 7.8 g | 342.6 ± 10.3 g | 359.6 ± 18.4 g |

[a]mean ± standard deviation, N=9

[b]One-way ANOVA of rats administered SRBC on Day 0 showed significant effect ($p < 0.05$) of dose on Day 6 rat weight. Tukey multiple comparisons test showed weight of $0.5 \times 10^8$ cells/rat group significantly greater ($p < 0.5$) than $1 \times 10^8$ cells/rat group.

Table S2 Male spleen weights from sheep red blood cell (SRBC) optimization study. Rats were administered SRBC on Day 0. Rats were euthanized on Day 6 and spleens were removed and weighed.

| Dose (SRBC/rat) | Spleen Weight |
|---|---|
| $0.5 \times 10^8$ | 0.93 ± 0.19[a,b] g |
| $1 \times 10^8$ | 0.80 ± 0.23 g |
| $2 \times 10^8$ | 0.79 ± 0.07 g |

[a]mean ± standard deviation, N=9

[b]One-way ANOVA of spleen weight data showed no effect of dose.

Table S3 IgM (Units/mL) in male rat serum following exposure to sheep red blood cells (SRBC). Rats were administered SRBC on Day 0. Blood was withdrawn on Days 4, 5 and 6. IgM was quantified in serum.

| Dose (SRBC/rat) | Day 4 | Day 5[b] | Day 6[b] |
|---|---|---|---|
| $0.5 \times 10^8$ | 5619.4 ± 5071.4[a] | 25476.7 ± 27213.3 | 37170.7 ± 36723.7 |
| $1 \times 10^8$ | 10480.3 ± 13416.5 | 29393.5 ± 36429.7 | 31343.1 ± 41549.1 |
| $2 \times 10^8$ | 15871.2 ± 16028.3 | 48996.8 ± 51342.3 | 50447.9 ± 47646.4 |

[a]mean ± standard deviation, N=9

[b]Two-way ANOVA of serum IgM in rats administered SRBC showed significant effect of Day ($p < 0.01$). Tukey's multiple comparisons test showed serum IgM levels were significantly greater on Day 5 ($p < 0.05$) and Day 6 ($p < 0.01$) than Day 4.

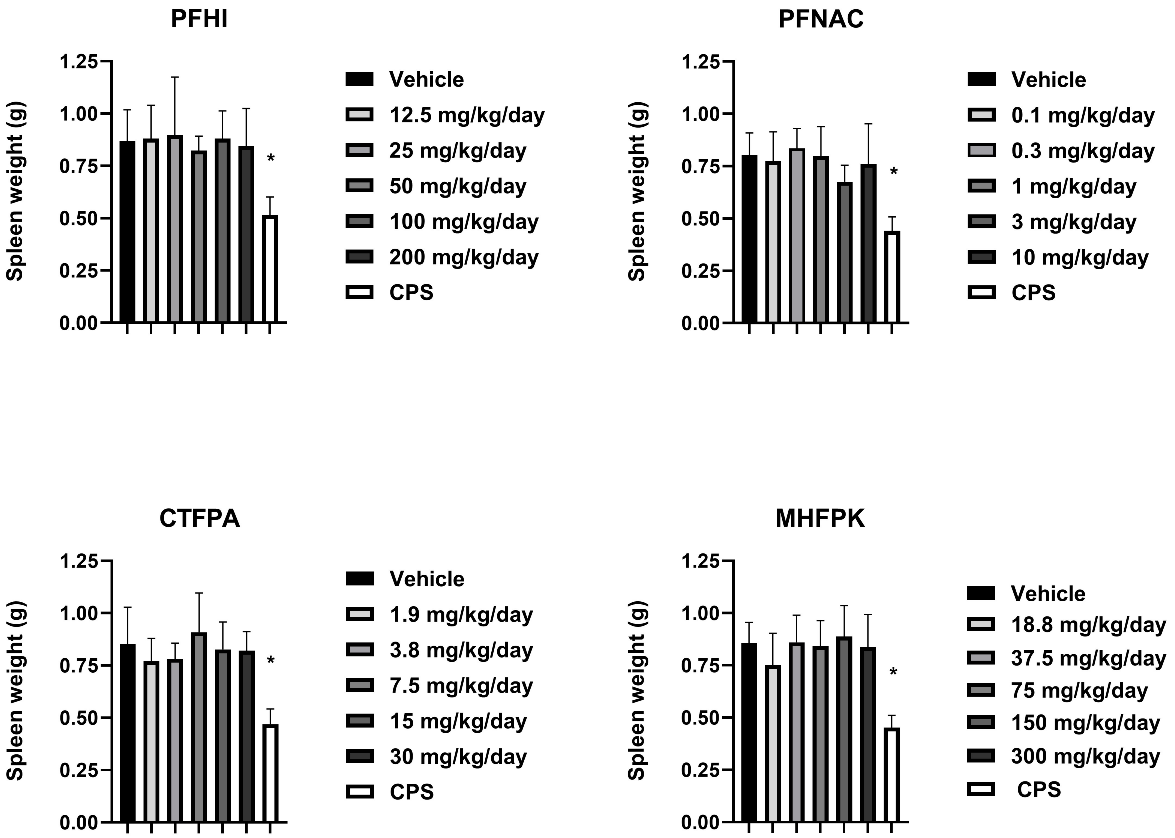


Figure S1 Spleen weight (g) of rats dosed by oral gavage daily from Day 0–27 with PFHI, PFNAC, CTFPA, or MHFPK. Vehicle control was corn oil. Positive control rats were administered CPS ip in sterile saline on Days 22–28. Rats were administered the antigen SRBC iv on Day 22. Rats were euthanized on Day 28. Data represents mean ± SD, N=12/dose group, except for 200 mg/kg/day PFHI dose group, N=11. *Significantly different from vehicle control, $p < 0.05$.

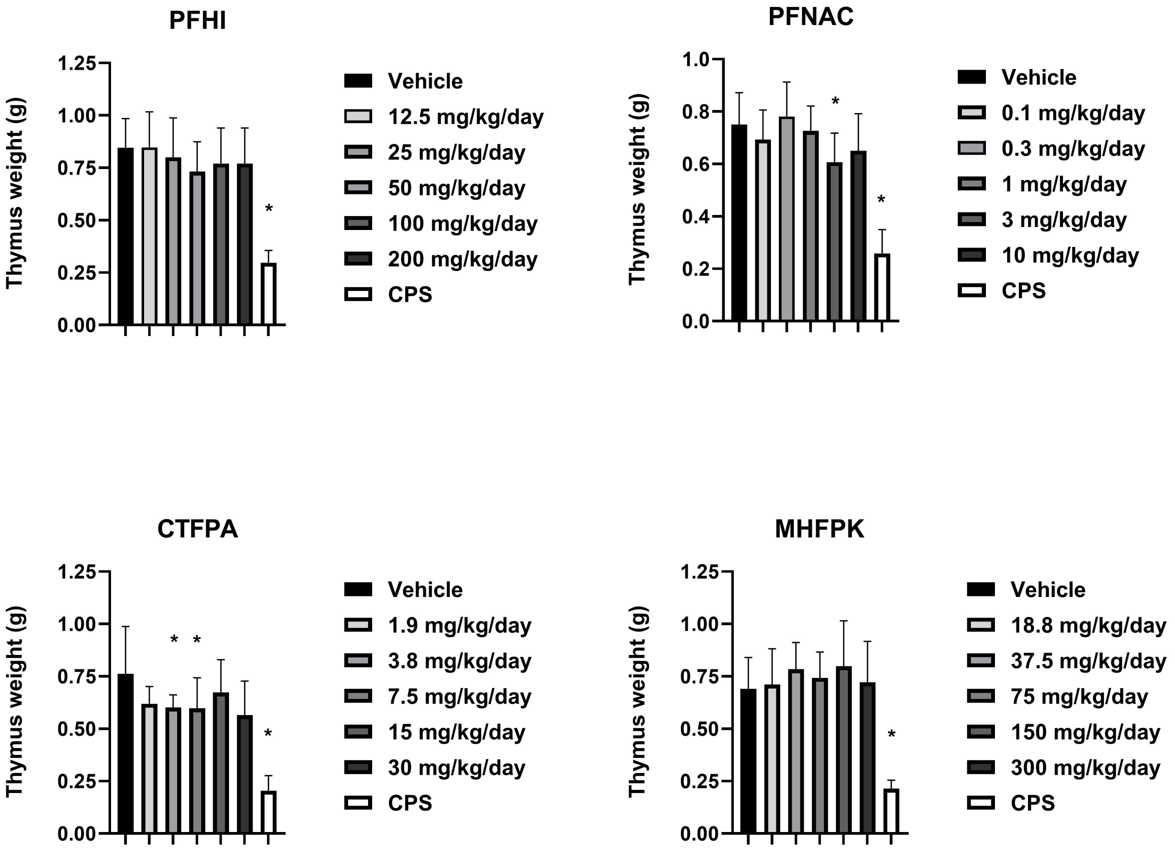


Figure S2 Thymus weight (g) of rats dosed by oral gavage daily from Day 0–27 with PFHI, PFNAC, CTFPA, or MHFPK. Vehicle control was corn oil. Positive control rats were administered CPS ip in sterile saline on Days 22–28. Rats were administered the antigen SRBC iv on Day 22. Rats were euthanized on Day 28. Data represents mean ± SD, N=12/dose group, except for 200 mg/kg/day PFHI dose group, N=11. *Significantly different from vehicle control, $p < 0.05$.
